# Oncogenic micro-RNAs and Renal Cell Carcinoma

**DOI:** 10.3389/fonc.2014.00049

**Published:** 2014-03-17

**Authors:** Cristina Grange, Federica Collino, Marta Tapparo, Giovanni Camussi

**Affiliations:** ^1^Department of Medical Sciences, University of Torino, Torino, Italy; ^2^Translational Center for Regenerative Medicine, University of Torino, Torino, Italy

**Keywords:** cancer stem cells, circulating miRNAs, EMT transition, RCC biomarkers, tumor plasticity, kidney

## Abstract

Tumor formation is a complex process that occurs in different steps and involves many cell types, including tumor cells, endothelial cells, and inflammatory cells, which interact to promote growth of the tumor mass and metastasization. Epigenetic alterations occurring in transformed cells result in de-regulation of miRNA expression (a class of small non-coding RNA that regulates multiple functions), which contributes to tumorigenesis. The specific miRNAs, which have an aberrant expression in tumors, are defined as oncomiRNAs, and may be either over- or under-expressed, but down-regulation is most commonly observed. Renal cell carcinoma (RCC) is a frequent form of urologic tumor, associated with an alteration of multiple signaling pathways. Many molecules involved in the progression of RCCs, such as HIF, VEGF, or mammalian target of rapamycin, are possible targets of de-regulated miRNAs. Within tumor mass, the cancer stem cell (CSC) population is a fundamental component that promotes tumor growth. The CSC hypothesis postulates that CSCs have the unique ability to self-renew and to maintain tumor growth and metastasis. CSCs present in RCC were shown to express the mesenchymal stem cell marker CD105 and to exhibit self-renewal and clonogenic properties, as well as the ability to generate serially transplantable tumors. The phenotype of CSC has been related to the potential to undergo the epithelial–mesenchymal transition, which has been linked to the expression pattern of tumorigenic miRNAs or down-regulation of anti-tumor miRNAs. In addition, the pattern of circulating miRNAs may allow discrimination between healthy and tumor patients. Therefore, a miRNA signature may be used as a tumor biomarker for cancer diagnosis, as well as to classify the risk of relapse and metastasis, and for a guide for therapy.

## OncomiRNAs

miRNAs are a class of small non-coding RNAs involved in multiple biological processes. Generation of mature miRNAs requires several steps. Pri-miRNA is the first inactive form produced within the nucleus. Pri-miRNA forms a stem-loop structure recognized by a protein complex, composed of DGCR8 and Drosha enzyme. This protein complex processes the pri-miRNA into a double-stranded hairpin structure of approximately 70 nucleotides, which is called precursor miRNA (pre-miRNA) (1, 2). These initial steps all take place in the nucleus, followed by exportation of the pre-miRNA to the cytoplasm in a GTP-dependent manner, mediated by another protein complex, Exportin-5 bound to Ran (3). In the cytoplasm, the pre-miRNA is further processed to generate a dsRNA by the RNase III enzyme, Dicer. One of the two strands that are generated, known as passenger miRNA or miRNA*, is degraded. The remaining mature single-stranded (ss) miRNA, associates with the RNA-induced silencing complex (RISC). The resulting complex is then able to bind the 3′-untranslated region (3′-UTR) on the target mRNA.

miRNAs interact with the region recognized in the mRNA sequence, by two to eight nucleotides present at the 5′ end of the miRNA. This region is called the seed sequence and determines the specificity of the interaction with the mRNA at its 3′-UTR (4). If the miRNA/mRNA interaction is precise, the mRNA is immediately degraded by Ago2, a protein of the argonaute family. Conversely, if there is no precise interaction, a translational repression of the mRNA occurs (4).

Abnormally expressed miRNAs in cancer are called oncomiRNAs. Numerous evidence correlates miRNA de-regulation with tumor stages, types, and progression. The epigenetic alterations that usually take place in a transformed cell, are probably responsible for the miRNA de-regulation observed in cancer (5). miRNA expression and cancer progression are usually inversely correlated. Indeed, an overall down-regulation of miRNAs is usually observed in tumor cells, although there may also be a small group of pro-oncogenic miRNAs that is up-regulated. Recently, the possible therapeutic use of miRNAs has been evaluated. This may include miRNA replacement therapies for tumor-suppressor-miRNAs, and inhibition strategies for oncogenic miRNAs (5).

## Cancer Stem Cells and OncomiRNAs

The cancer stem cell (CSC) hypothesis postulates that there is a rare subpopulation of cancer cells that possesses the ability to self-renew and sustain tumor growth and metastasis. CSCs divide asymmetrically, generating one identical daughter cell and one cell that starts to differentiate and divide (6). The presence of CSCs was first described in 1997 by Bonnet and colleagues (7), who identified CSCs in acute myeloid leukemia. Subsequently, in 2003, CSCs were also isolated in breast cancer by Al-Hajj et al. (8), using the specific cell surface markers, CD44 and CD24. Thereafter, CD133, CD166, and EpCAM, along with other surface markers, were also used to isolate CSCs in many tumors (9). During the last decade, CSCs were identified in numerous solid tumors, such as ovary, brain, pancreatic, prostate, colon, melanomas, and hepatocellular cancers (6). Functional criteria for CSC identification include clonogenicity and the ability to generate serially transplantable tumors, starting from a very low number of cells (10).

miRNAs may regulate the properties of CSCs by acting on several different signaling pathways. The progression from a stem cell to a terminally differentiated cell depends on a temporal balance between proliferation and differentiation programs. This balance is altered in tumors, partly as consequence of miRNA de-regulation, leading to the maintenance of proliferation and self-renewal of CSCs (11). miRNAs are divided in two categories based on their effect on stem cell phenotype, namely miRNAs that play a role in pluripotency, and miRNAs involved in differentiation. The first group controls the capacity of self-renewal and cell division, and inhibits the differentiation of stem cells. miR-184, -137, -290, -302, -200, and -9 all belong to this class (12). On the other hand, miRNAs that promote differentiation are let-7, miR-134, -122, -181, -145, -296, and -470 (12). miR-134, -296, and -470 suppress the self-renewal ability of CSCs by targeting some of the main molecules involved in the stem cell phenotype, such as Oct-4, Nanog, and Sox2 (12).

The first evidence that miRNA expression was down-regulated in CSCs was shown in breast CSCs. Yu et al. demonstrated the decrease of let-7, miR-16, -107, -128, -20b and of all the members of the miR-200 family (13). Within this group of miRNAs, the main miRNA involved in the suppression of CSC self-renewal was shown to be let-7 (12).

Let-7 is a ubiquitously expressed family that has been highly conserved through evolution, suggesting a pivotal role for these miRNAs in the regulation of proliferation and differentiation; this family was one of the first mammalian groups of miRNAs ever discovered (14). Let-7 miRNAs are regulators of cell cycle exit and differentiation, and their targets are cell cycle modulators, such as CDC25A and CDK6 and different early embryonic genes (14, 15). There is a reduced expression of let-7 miRNAs in CSCs of different tissues, such as gastric and breast carcinomas (13, 16). Over-expression of this family in tumor cells was shown to induce reduction of tumor progression and inhibition of stemness properties (17). Moreover, let-7 members have been classified as negative regulators of the epithelial-to-mesenchymal transition (EMT) (17).

Of those miRNAs defined as tumorigenic, miR-21 plays an important role. An increased expression of miR-21 was observed in many tumors, including prostate, brain, breast, and pancreatic cancer, and correlates with a poor patient prognosis (18).

Another family of miRNAs that is often down-regulated during cancer progression is miR-200 and is composed of five members (miR-200a, -200b, -200c, -141, and -429). miR-200a, b, and c are significantly decreased in CSCs derived from human and murine breast cancer (19).

Despite the huge number of miRNAs that are commonly altered during tumor development, some of them are considered to be tumor specific. For example, miR-22 acts as a tumor suppressor during liver carcinogenesis, and its down-regulation is associated with a poor clinical outcome. Experimental results have shown that the over-expression of miR-22 leads to a reduction of tumor cell invasion and growth affecting p53, p21, and PTEN (17). In addition, CSCs from hepatocellular carcinoma are characterized by the up-regulation of miR-92, -93, -181 family, -17, -20a, -25, and -106b (20).

miR-101, -26, and -34a are additional examples of miRNAs with tumor suppressor functions, and are down-regulated in many solid cancers, including hepatic, pancreatic, lung, and prostate cancers (17).

Recent studies have suggested a connection between CSCs and the EMT. The EMT is an evolutionary-conserved biological process whereby epithelial cells acquire mesenchymal characteristics, and is involved in apoptosis resistance, tumor motility, and invasion. Molecules associated with this process are transcription factors, such as ZEB (ZEB1 and 2), Snail, Slug, and Twist1, which act by repressing E-cadherin and inducing vimentin and fibronectin expression (21). The EMT process is strongly regulated by miRNAs; for example, the miR-106b-25 cluster has been shown to induce the EMT and CSC phenotype in human breast cancer cells, acting downstream of the transcription factor Six1. A direct target of this miRNA family is the Smad7 protein; reduction of Smad7 leads to activation of TGFβ signaling, which regulates the EMT (22). Mani et al. (23) recently demonstrated the down-regulation of miR-203 in CSCs that were undergoing the EMT (23). Re-expression of miR-203 compromises the cell migratory and invasive capacity *in vitro* and tumor initiation and metastasis *in vivo*.

## Role of miRNAs in Metastasis

Metastasization is a tumor-specific mechanism divided into different steps. Tumor cells invade the extracellular cell matrix (ECM) in order to access the blood fluid, after which they extravasate at distant sites where they form secondary tumors. The role of miRNAs in the acquisition of metastatic phenotypes has been well established. miRNAs involved in metastasization are either classified as metastasis-suppressive or metastasis-promoting miRNAs (24).

The first event that occurs in metastasization is the enhanced capability of tumor cells to invade the ECM through destruction of ECM proteins, and regulation of this process is carried out by metastasis-promoting miRNAs. The invasive potential of prostate cancer cells has been shown to be controlled by miR-21, which targets MARCKS, involved in cell motility (25). Moreover, miR-21 has been associated with tumor cell invasion and metastasization in colorectal and breast cancers, where it targets tumor suppressor PDCD4 at a post-transcriptional level (26). In addition, miR-21 has been correlated with the ability of glioma cells to migrate and invade, as a consequence of modulation of the metalloproteinase inhibitors, RECK and TIMP3 (27).

Another example of a metastasis-promoting miRNA is miR-10b, which has been described as a metastatic promoter in breast cancer cells. miR-10b down-regulates the metastatic suppressive gene, homeobox D10, which in turn inhibits the metastasis-promoting gene *RHOC* (28). miR-373 and -520c, which belong to the same family as miR-10b, have also been classified as pro-metastasis miRNAs (29). The target of this particular miRNA family was found to be CD44, and its down-regulation has been associated with the acquisition of an enhanced migratory potential (29). Similarly, miR-182 over-expression promotes migration and invasion in melanoma cells (30). miR-30b/30d also correlates with tumor melanoma progression via down-regulation of GALNT1 and GALNT7, which are suppressors of cell invasion (31). In addition, miR-126 and -183 over-expression has been observed to be involved in the metastasization of lung cancer (32, 33).

Metastasis-suppressive miRNAs (24) were first identified in breast cancer cells (34). Over-expression of miR-335, -126, and -206 was shown to block the ability of tumor cells to invade and generate metastasis in bone and lungs (34). On the contrary, down-regulation of miR-335 by antagomirs enhances metastasis formation (34), and miR-335 plays a regulatory role in the expression of a set of metastatic genes, such as *Tenascin-C* and *SOX4* (35, 36). On the other hand, miR-126 acts principally to inhibit tumor growth, endothelial activation, and metastatic initiation (37, 38), whilst miR-31 has been described to inhibit cancer progression and metastasization in breast tumors (39). The cohort of pro-metastatic target genes affected by miR-31 expression includes *Integrin-alpha V*, *RDX*, and *RHOA*, all involved in cell migration (40). Another metastasis-suppressive miRNA, miR-146b, which targets the matrix metalloproteinase 16, was shown to reduce the invasion potential of glioblastoma U373 cells (41). miR-205, has also been shown to promote E-cadherin expression and reduction of prostate cancer cell migration and invasion (42).

## Circulating Tumor-Derived miRNAs

Circulating RNAs in body fluids were first described in 1972 by Kamm and Smith (43). Circulating miRNAs in body fluids are present as either vesicle-encapsulated or non-encapsulated. Extracellular vesicles (EVs) are circular membrane fragments of different origins that retain characteristics of the cell of origin, and contain biological materials (lipids, proteins, and genetic information) (44). EVs are described as a mechanism for cell-to-cell communication, capable of influencing the phenotype of target cells. The EV signaling mechanism can occur by different ways, for example, direct stimulation via surface-receptors, or transfer of proteins or genetic materials, such as mRNAs and miRNAs (45). Many authors have described the presence of miRNAs in biological fluids that are contained within EVs, and confer resistance to RNAse activity and persistence within the circulation (46–48). Non-encapsulated miRNAs that are present in serum and plasma are associated with proteins such as lipoproteins and Ago family proteins (49–51). It was recently discovered that miRNAs are present at high levels in the blood stream of cancer patients (47, 52). The detection and characterization of circulating miRNAs can be a powerful tool for non-invasive diagnosis of different cancers (53–55). Lawrie et al. (52) described circulating miRNAs in the serum of B-cell lymphoma patients, and they were able to discriminate between healthy controls and patients with tumors by screening for three tumor-associated miRNAs (miR-155, -210, and -21).

Further investigations into the presence of circulating miRNAs were carried out by Mitchell et al. (47), using a prostate tumor xenograft mouse model. They observed that human miRNAs are detectable in the bloodstream when the tumor is well established. By screening a list of candidate miRNAs present in the plasma, the authors proposed miR-141 as a prostate cancer biomarker, and by analyzing miR-141 expression, they were able to distinguish between healthy controls and advanced prostate cancer patients.

In a recent study, seven miRNAs (miR-10b, -21, -125b, -145, -155, -191, and -382) were found to be up-regulated in breast cancer patients, and were validated using receiver operating characteristic (ROC) curves to determine the specificity and sensitivity. Analysis showed that only three of these miRNAs were found to be related to breast cancer progression (56). In addition, miR-210 was found to be strongly increased in the serum of patients with circulating tumor cells, in metastatic breast cancer (57). Conversely, another study demonstrated a reduction in circulating miRNAs in cancer patients (58). In plasma vesicles of lung cancer patients, Silva et al. (58) observed a reduction in let-7f, miR-20b, and miR-30e-3p. Let-7f and miR-30e-3p levels facilitating the distinction between different lung cancer stages, suggesting that they may be prognostic biomarkers.

Circulating miRNAs may also discriminate between patients with and without metastasis. For example, in breast cancer, miR-10b, -34a, and -155 correlate with the presence of metastases (59). In addition, in prostate cancer, five miRNAs (miR-375, -9*, -141, -200b, and -516a-3p) are associated with the incidence of metastases (60).

## miRNA Profile in Different Renal Cell Carcinoma Subtypes

Renal cell carcinoma (RCC) is a frequent form of urologic tumor, and represents 3% of total human cancers, with a high index of relapse and a mortality rate of over 40%. During the last 30 years, the incidence of RCC has increased globally, and has became the seventh most common carcinoma in men and the eighth most common in women in the USA (61). RCC is composed of many different subtypes such as clear cell, papillary, chromophobe, and collecting duct carcinomas (62), which differ in their clinical outcome and biological features. The majority (75–80%) of renal tumors are clear cell RCC (ccRCC). This cancer type is highly vascularized due to mutation or hypermethylation of the onco-suppressor gene, Von-Hippel–Lindau (VHL). Alteration of the VHL protein causes constitutive activation of the angiogenic process, by means of *HIF1/HIF2* gene regulation (63).

The correct diagnosis for each type of RCC is fundamental for the outcome of the patient because each subtype behaves differently in terms of prognosis and response to treatment. Traditional diagnostic approaches, based on the histopathological profiles, have been improved with innovative techniques. These include the development of new biomarkers that could discriminate tumor from normal tissue and identify tumor subtypes. In this contest, miRNA expression profile may provide new diagnostic approaches (64, 65).

Lu et al. (66) demonstrated the possibility to use miRNAs for the identification of human cancers with higher accuracy compared with mRNAs, suggesting miRNAs as good candidates as biomarkers. Whereas miRNAs allowed classification of poorly differentiated tumors, mRNA profile did not (66). Recently Youssef et al. (67) developed a classification able to discriminate the different RCC subtypes, by comparison of relative expression of specific miRNA pairs in a four-step decision tree. They analyzed 94 different cases of freshly frozen tissues by microarray and identified 15 differentially expressed miRNAs (miR-126, -192, -194, -200b, -221, -222, -182, -548m, -183, -663, -22, -498, -25, -200c, -21). Hierarchic clustering showed similarity between ccRCC and papillary RCC (pRCC) and difference of ccRCC and pRCC in respect to oncocytoma and chromophobe RCC (chRCC). This system provided 97% sensitivity to discriminate normal from RCC and 100% sensitivity to distinguish different RCC subtypes. Similar results have been described by other groups (64, 68). In particular, the increased level of miR-200b in chRCC compared with oncocytoma was also found by Petillo et al. (68). A similar methodology, using a miRNA marker algorithm, was used by Fridman et al. (64) to classify RCC, with an accuracy of 90% compared with traditional diagnosis. In this study, the authors reported an up-regulation of miR-221 in chRCC and oncocytoma and not in ccRCC and pRCC (64).

## Alteration of miRNA Expression Levels in RCC

**Figure 1:**
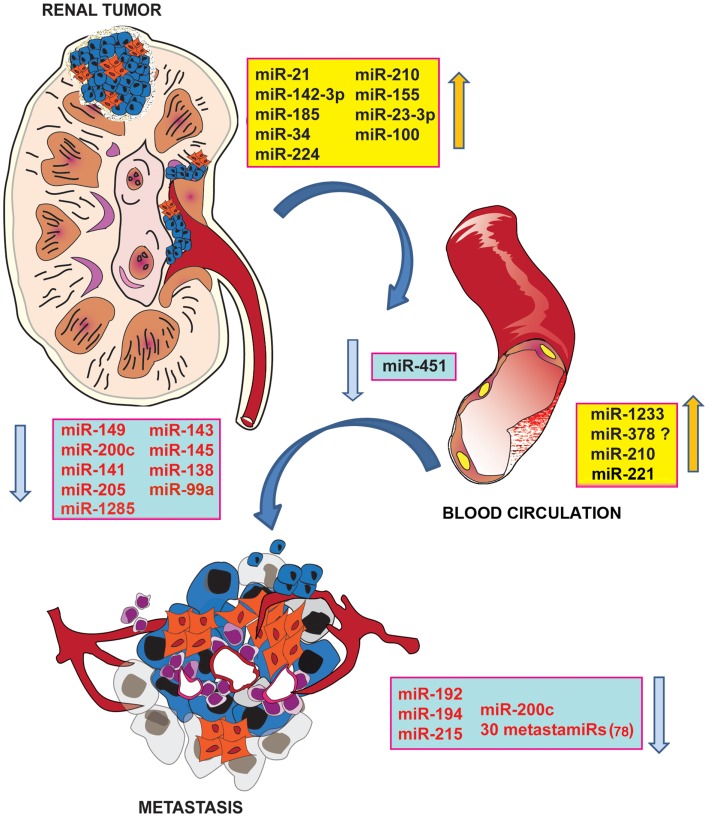
**De-regulated miRNAs in RCCs**. Schematic representation of up- and down-regulated miRNAs in primary and metastatic RCCs and in serum of RCC patients. miRNA profile has been suggested as a new accurate method for the identification of human cancer stages and subtypes. Screening of miRNAs in RCC samples in respect to normal kidney identified groups of oncogenic miRNAs (in blue), which act via down-regulation of tumor suppressor genes and anti-tumor miRNAs (in yellow), which target onco-proteins. miRNAs have been also correlated to the metastasization process. miRNA signature in metastatic RCC samples compared with primary RCC showed a significant reduction of some miRNAs (in yellow), targeting molecules involved in the EMT transition and invasion. miRNAs have been detected in serum of RCC patients, supporting their suitability as biomarkers.

The comparison of the expression levels of miRNAs in RCC with those of normal kidneys was evaluated in many studies, leading to the generation of a list of down-regulated and up-regulated miRNAs (Figure 1). Up-regulated miRNAs include miR-210, -155, -21, -142-3p, -185, -34, and -224, which function by down-regulating tumor suppressor genes (69). miR-210 and -155 instead are known to be related to HIF molecules and hypoxia (70). On the other hand, the list of down-regulated miRNAs includes miR-149, -200c, and -141, the loss of which leads to activation of oncogenes (69). In particular, miR-141 and -200c are members of the miR-200 family, which are often switched off in different tumors, and are associated with the EMT (71).

Hidaka et al. (72) focused the attention on down-regulated miRNAs in RCC and generated a list of 103 miRNAs. By a functional approach, 14 miRNAs were validated as tumor suppressors in RCC (miR-1285, -206, -1, -135a, -429, -200c, -1291, -133b, -508-3p, -362-3p, -509-5p, -218, -335, -1255b, and -141) (72). Some of them (miR-1-1/-133a-2, miR-1-2/-133a-1, and miR-206/-133b) were found in specific chromosomal regions of human genome, known to be often down-regulated in different human cancers (72).

Many molecules involved in the progression of RCCs, such as HIF, VHL, VEGF, or mammalian target of rapamycin (mTOR), are possible targets for de-regulated miRNAs (73). Therefore, several studies have focused on the biological implication of up- or down-regulated miRNAs in RCC.

miR-21 is a tumorigenic miRNA, involved in the progression and in metastasization of many solid tumors as we described above. Recently, a genomic study on ccRCC defined by the Cancer Genome Atlas, demonstrated the correlation of miR-21 with a worst survival and the connection with a shift toward a “Warburg effect”-like state. In fact, a decreased methylation on the promoter of miR-21 (that means higher expression) was highlighted being clearly associated with worst outcome (74). Moreover, miR-21 expression was induced by high glucose levels and was able to down-regulate PTEN (18). In addition, increased expression of miR-21 has been shown to alter the expression of many proteins involved in tumor invasion (18).

Zaman et al. (75) demonstrated that also miR-23-3p is an oncogenic miRNA targeting PTEN and its expression level was increased in RCC. Wang et al. showed a correlation of miR-100 up-regulation with poor prognosis in RCC patients (76). Conversely, suppression of miR-205 has been described as being associated with activation of the ERK1/2 pathway and of FAK and STAT3 proteins (77). Of particular interest, miR-1285 has been identified as a tumor-suppressor-miRNA. When restored in renal tumor cells, miR-1285 significantly inhibited cell proliferation, invasion, and migration (72). Hikada and colleagues demonstrated that transglutaminase 2 (TGM2) is one of the targets of miR-1285. Increased expression of TGM2 has been shown in many tumors and has been correlated with EMT, drug resistance, and metastasis (72).

Another important example of down-regulation of tumor-suppressor-miRNAs is miR-138 that targets vimentin (78). Vimentin participates to cancer cell migration, metastasis, and invasion and it is overexpressed in human specimens of RCC. In addition, the positive staining for vimentin in combination with CD9 has been proposed as a marker to distinguish ccRCC and chRCC (79). The significant decrease of miR-138 was also highlighted, by Girgis et al. (80) who performed an integrated analysis of copy number, gene expression (mRNAs and miRNAs), protein expression, and methylation changes in ccRCC. miR-138 is located on the 3p arm, a region that is the most frequently deleted in ccRCCs. Other important examples of down-regulation of tumor-suppressor-miRNAs are the reduced expression of miR-143 and -145, which target hexokinase-2 (81). miR-99a is down-regulated in several human malignancies. Cui et al. (82) evaluated its expression and its role in RCC by RT-PCR, demonstrating that the level of miR-99a was significantly decreased in tumor samples compared with adjacent tissues. In addition, low expression level of miR-99a was correlated with poor survival of RCC patients. The restoration of miR-99a on renal cancer cell lines suppressed tumorigenic properties of cells mainly arresting them in G1-phase *in vitro*. Intra-tumoral delivery of synthetic miRNA, in an *in vivo* model of human tumor, was shown to block tumor progression. The target of miR-99a is the mTOR, a molecule often activated and altered during cancer progression and metastasis (82).

## Metastasis in RCC and miRNA Profile

The presence of metastasis in RCC patients is associated with a mortality of 90% (83). Analysis of the miRNA signature in metastatic RCC compared with primary RCC showed a significant reduction in miR-192, -194, and -215 (Figure 1). Targets of these miRNAs are ZEB2 and MDM2, molecules associated with the EMT (83). In addition, miR-200c, which targets ZEB1, is markedly decreased in metastatic RCC (84). Wotschofsky et al. (85), by comparing the miRNA expression profile of primary ccRCC with metastasis, generated a list of 30 down-regulated miRNAs in metastatic ccRCC, which have thus been defined as metastamirs (Figure 1). The aim of this study was to evaluate the differential expression of miRNAs among normal, primary tumor, and metastatic tissue using microarray screening and subsequent validation by RT-PCR (85). A parallel study, demonstrated a correlation between a specific miRNA signature and early tumor relapse after nephrectomy (65). Sixty-four miRNAs have been found to be differentially expressed between relapse-free RCC patients and RCC patients who developed relapse. The expression level of miR-143, -26a, -145, -10b, -195, and -126 was low in tumors from patients who developed relapse and in primary metastasis. For this reason, miR-145, -126, and -127-3p have been proposed as biomarkers for relapse-free survival (65). Some of these miRNAs, such as miR-143, -145, and -26a have been described as tumor-suppressor-miRNAs in many human tumors (65). The same approach was used by Wu et al. (86) with the aim to identify a miRNA signature to predict the risk of metastasis. The expression level of miR-10b, -139-5p, -130b, and -199b-5p was altered in primary ccRCC and correlated with the status of ccRCC metastasis. Thus, these miRNAs have been proposed as prognostic factors for the development of metastasis (86).

## Profile of Circulating miRNAs in Renal Cell Carcinoma

Only few studies have investigated circulating levels of miRNAs in RCC (Table 1). In some of these studies, the authors compared the miRNA expression profile in serum samples with that of tissues (Figure 1). Wulfken et al. (87) observed the up-regulation of 109 miRNAs in serum samples of RCC patients, 36 of which were present at high levels both in tissue and serum samples. Among the validated miRNAs, the authors proposed miR-1233 as a RCC marker that provides 77% sensitivity but only 37.6% specificity. Redova et al. (88) described 30 miRNAs differentially expressed in the serum of RCC patients with respect to healthy controls; of which 19 miRNAs were up-regulated and 11 miRNAs were down-regulated. Two of these miRNAs have been successfully validated as potential biomarkers, namely miR-378, which is increased, and miR-451, which is decreased, in the serum of RCC patients. At variance to these results, in a recent paper, the attempt to validate miR-378 in the serum of ccRCC patients failed. This particular study concludes that miR-378 is not able to discriminate between RCC patients and control subjects (89). Possible explanations for these differences in study results could be the sample source and storage as well as the processing and selection of internal controls.

**Table 1:** **Circulating miRNAs in RCC**.

miRNAs	Sample sources	Analysis method	Normalization method	Reference
miR-1233	RCC serum vs. RCC tissue	TaqMan low density array	cel-miR-39	Wulfken et al. (87)
miR-378	RCC serum vs. healthy control	TaqMan low density array	miR-16	Redova et al. (88)
miR-451	
miR-378	RCC serum vs. healthy control	TaqMan low density array	cel-miR-39	Hauser et al. (89)
miR-210	RCC serum vs. RCC tissue	qPCR	5s rRNA	Zhao et al. (90)
miR-15a	Urine	qPCR	β-actin	von Brandenstein et al. (93)

The up-regulation of HIF, due to mutation of VHL, directly regulates miR-210 expression. Zhao et al. (90) recently proposed miR-210 as a novel biomarker for RCC. They observed that miR-210 was expressed at high levels in RCC serum, but decreased 7 days after surgery.

Finally, Teixeira et al. (91) correlated the expression levels of circulating miR-221/222 and the patients’ overall survival. High levels of miR-221 have been associated with metastasis and with a significant reduction of overall survival. Based on these results, miR-221 has been proposed as potential biomarker of RCC progression (91).

Urine is another bio-fluid suitable for investigating RCC biomarkers (92). Only one study describes miRNAs as biomarkers in urine of RCC patients. von Brandenstein et al. (93) observed an up-regulation of miR-15a in the urine of RCC patients compared with healthy subjects. The expression of miR-15a is regulated by PKC, which blocks the release of the primary miRNA by direct binding. The expression pattern of PKC isoforms differs between RCC and benign oncocytoma; in fact, PKC is up-regulated in benign oncocytoma but down-regulated in RCC. Conversely, miR-15a is up-regulated in RCC and down-regulated in oncocytoma suggesting that this miRNA may be a marker of ccRCCs (93).

In RCC, a small population of cells expressing the mesenchymal stem cell marker CD105 has been identified as CSCs. This population grows as spheres, possesses clonogenic ability, and expresses Nestin, Nanog, and Oct-3/4 stem cell markers (94). CD105^+^ renal CSCs show the unique ability to generate serially transplantable tumors *in vivo* that resemble the tumor of origin (94). We found that EVs derived from CD105^+^ renal CSCs may modify the tumor microenvironment. These EVs, at variance to those derived from differentiated tumor cells, are able to trigger angiogenesis and to induce the formation of a pre-metastatic niche in the lung (95). Comparison of RNAs present inside these two different types of vesicles showed the enrichment of transcripts coding for several pro-angiogenic proteins such as VEGF, FGF, angiopoietin 1, ephrin A3, MMP2, and MMP9 in EVs derived from CSCs. The miRNA expression profile of EVs released from CSCs and differentiated tumor cells was also compared. This analysis revealed the presence of 24 significantly up-regulated miRNAs and 33 down-regulated miRNAs in CSC-derived EVs (95). Prediction for targets of miRNA that are enriched in CSC-derived EVs by gene ontology analysis, illustrated a strong over-representation of crucial biological processes like transcription, metabolic processes, nucleic acid binding, cell adhesion molecules, and regulation of cell proliferation. Among the miRNAs shuttled by CSC-derived EVs, we detected miR-200c, -92, and -141, which have been described as significantly up-regulated in patients with ovarian, colorectal, and prostate cancer, respectively (95). In addition, miR-29a, -650, and -151 were associated with tumor invasion and metastases (96–98). Moreover, the enrichment of miR-19b, -29c, and -151 has been described as being directly associated with RCC (99).

## miRNA Targeting and Therapy

The evidence that some miRNAs are commonly de-regulated in many human cancer leads to the hypothesis to exploit them as new therapeutic targets. The inhibition of oncogenic miRNAs is a promising approach that can be achieved through siRNA- or antisense oligonucleotide-based therapy (100). This strategy has been set up in different xenograft tumor models; for example, an antisense oligonucleotide targeting miR-21 has been successfully used *in vitro* and *in vivo* in a breast cancer model (101). Kim et al. (102) used a miRNA-221 molecular beacon (miR-221-MB)-conjugated magnetic/fluorescence nanoparticle probe as a cancer-targeting theranostic dye (MFAS-miR-221-MB) to simultaneously follow localization and inhibition of miR-221 in thyroid cancer.

Another approach used to decrease the level of oncomiRNAs is the use of miRNA sponges. This strategy consists in cloning multiple copies of a specific sequence, complementary to a selected miRNA. miRNA sponges, once transfected into tumor cells, compete with the natural target for miRNA binding. Using this technique, Ma et al. (103) demonstrated a reduction of invasiveness of a breast cancer cell line using a sponge trapping miR-9.

On the other hand, tumor-suppressor miRNAs are usually down-regulated in cancer and the administration of synthetic miRNAs may be a therapeutic option. This strategy is based on the replacement of the under-represented miRNAs in the tumor. For example, the tumor suppressor let-7 has been injected intra-tumor in a mouse model of non-small cell lung cancer leading to the reduction of tumor size. Another miRNA often de-regulated in different cancers is miR-34. Recently, a miRNA-based formulation (MRX34) has entered in a clinical trial for hepatocellular carcinoma treatment. MRX34 is a liposomal nanoparticle loaded with a synthetic mimic of the tumor suppressor miR-34. In preclinical animal models, systemic administration of MRX34 has been shown to inhibit the growth of primary tumors, to block metastasis, and to extend survival (104).

## Conclusion

The presence of selected patterns of oncogenic miRNAs may be used as diagnostic and prognostic tool. The use of biological fluids such as serum, plasma, or urine may allow to perform non-invasive analysis. Circulating miRNAs are derived from different tumor populations (cancer cells, tumor-surrounding cells, and CSCs) and they could be also influenced by stages and progression of cancer.

The studies of miRNAs that are present in biological fluids and tissues have generated contrasting results according to technical procedures and analysis methods. One of the main problems is the stability of miRNAs in biological fluids due to the presence of degradative enzymes. Recent observations that EVs derived from tumor cells contain defined patterns of miRNAs may offer a new approach for the identification of miRNA biomarkers. In fact, vesicles are able to confer stability to extracellular RNAs.

Moreover, the heterogeneous sample sources (primary tumor tissue, metastasis tissue, healthy tissue, and serum) used to compare miRNA expression profile is another variable to take into account. Also, the choice of appropriate endogenous controls for data normalization is a relevant limit. The use of synthetic spike-in controls from *C. elegans*, such as cel-miR-39, -43, -54, and -238 has been proposed as an alternative normalizer instead of ubiquitary expressed miRNAs such as miR-16 or -21 (88, 105).

However, some miRNAs, present in biological samples, have been proposed as prognostic factors even if, at the moment, they are not validated in preclinical and clinical setting. Table 2 summarizes candidate miRNAs proposed as prognostic biomarkers for RCC.

**Table 2:** **Prognostic biomarkers for RCC**.

Primary tumor		Metastasis		Poor overall survival	
miRNA	Biological sample	miRNA	Biological sample	miRNA	Biological sample
miR-210 (90)	Serum	miR-221 (91)	Serum	miR-221 (91)	Serum
miR-210 (69)	Tumor tissue	miR-10b, -139-5p, -130b, -199-5p (86)	Primary tumor tissue	miR-100 (76)	Tumor tissue
miR-21 (106)	Tumor tissue			miR-21 (74)	Tumor tissue

In conclusion, the use of miRNAs as biomarkers in clinical practice is a potentially powerful tool for non-invasive analysis, which, at the moment requires further development.

## Conflict of Interest Statement

The authors declare that the research was conducted in the absence of any commercial or financial relationships that could be construed as a potential conflict of interest.
